# A Phox2b BAC Transgenic Rat Line Useful for Understanding Respiratory Rhythm Generator Neural Circuitry

**DOI:** 10.1371/journal.pone.0132475

**Published:** 2015-07-06

**Authors:** Keiko Ikeda, Masanori Takahashi, Shigeru Sato, Hiroyuki Igarashi, Toru Ishizuka, Hiromu Yawo, Satoru Arata, E. Michelle Southard-Smith, Kiyoshi Kawakami, Hiroshi Onimaru

**Affiliations:** 1 Division of Biology, Hyogo College of Medicine, Nishinomiya, Hyogo, Japan; 2 Division of Biology, Center for Molecular Medicine, Jichi Medical University, Shimotsuke, Tochigi, Japan; 3 Department of Physiology, and Pharmacology, Tohoku University Graduate School of Medicine, Sendai, Miyagi, Japan; 4 Department of Developmental Biology and Neuroscience, Tohoku University Graduate School of Life Sciences and JST/CREST, Sendai, Miyagi, Japan; 5 Center for Biotechnology, Showa University, Shinagawa, Tokyo, Japan; 6 Division of Genetic Medicine, Department of Medicine, Vanderbilt University School of Medicine, Nashville, Tennessee, United States of America; 7 Department of Physiology, Showa University School of Medicine, Shinagawa, Tokyo, Japan; Seattle Children's Research Institute, UNITED STATES

## Abstract

The key role of the respiratory neural center is respiratory rhythm generation to maintain homeostasis through the control of arterial blood pCO_2_/pH and pO_2_ levels. The neuronal network responsible for respiratory rhythm generation in neonatal rat resides in the ventral side of the medulla and is composed of two groups; the parafacial respiratory group (pFRG) and the pre-Bötzinger complex group (preBötC). The pFRG partially overlaps in the retrotrapezoid nucleus (RTN), which was originally identified in adult cats and rats. Part of the pre-inspiratory (Pre-I) neurons in the RTN/pFRG serves as central chemoreceptor neurons and the CO_2_ sensitive Pre-I neurons express homeobox gene *Phox2b*. *Phox2b* encodes a transcription factor and is essential for the development of the sensory-motor visceral circuits. Mutations in human *PHOX2B* cause congenital hypoventilation syndrome, which is characterized by blunted ventilatory response to hypercapnia. Here we describe the generation of a novel transgenic (Tg) rat harboring fluorescently labeled Pre-I neurons in the RTN/pFRG. In addition, the Tg rat showed fluorescent signals in autonomic enteric neurons and carotid bodies. Because the Tg rat expresses inducible Cre recombinase in PHOX2B-positive cells during development, it is a potentially powerful tool for dissecting the entire picture of the respiratory neural network during development and for identifying the CO_2_/O_2_ sensor molecules in the adult central and peripheral nervous systems.

## Introduction

Respiration is an important function in animals and controlled by two main respiratory rhythm generators, the parafacial respiratory group (pFRG) and the pre-Bötzinger complex inspiratory group (preBötC), which reside in the ventral side of the medulla in rodents [1–4]. Although both generators can be independently active to produce intrinsic periodic respiratory bursts under specific conditions, they normally interact as a coupled oscillator system to regulate spontaneous respiratory rhythm [5–8]. The ventral part of the pFRG was identified electrophysiologically in a region ventral to the facial nucleus (nVII) and close to the ventral surface in neonatal rats [4]. This part of the pFRG overlaps with the retrotrapezoid nucleus (RTN), the latter was first identified in the adult cat by retrograde tracing analysis as a candidate site for respiratory rhythmogenesis and later in the adult rat [9–14]. We herein call it the "RTN/pFRG", which in rodents plays an important role in central chemoreception and respiratory rhythm generation [15–24].

The RTN/pFRG is positioned rostral to the preBötC [25], and the RTN/pFRG and preBötC contain different types of respiratory-related neurons, which were originally classified in terms of firing or discharging pattern, as well as sensitivity to neuromodulators [4, 25]. Among the respiratory neurons of the RTN/pFRG, some show special phasic discharge patterns and firing just before inspiration, and are thus referred to as pre-inspiratory (Pre-I) neurons. Recently, we have shown that the Pre-I neurons are the predominant neurons in the RTN/pFRG of neonatal rats, assemble around capillary blood vessels, express a paired-like homeobox 2b gene (*Phox2b*), and are postsynaptically CO_2_ sensitive [23, 24, 26]. The expression of *Phox2b* is a remarkable feature of Pre-I neurons and a useful marker for identifying the cytoarchitecture of neurons in the RTN/pFRG in the newborn rat medulla. Unlike the RTN/pFRG, neurons of the preBötC do not express *Phox2b* (Ikeda and Onimaru, unpublished results).

The *Phox2b* gene encodes a transcription factor, PHOX2B, that exhibits a unique expression pattern. *Phox2b* is expressed in an identical functional group of neurons involved in the regulation of the visceral reflex circuitry that controls the digestive, cardiovascular, and respiratory systems [27]. The *Phox2b*-positive neuronal circuits include afferent and efferent visceral neurons that form the sensory and motor arms and their nuclei in the central nervous system (CNS) (reviewed in [28]). In the peripheral nervous system (PNS), *Phox2b* is expressed in three epibranchial sensory ganglia; geniculate, petrosal, and nodose [29, 30]. *Phox2b* is also expressed in neural crest cell-derived autonomic ganglia (sympathetic, parasympathetic, and enteric), the adrenal medulla, and the carotid body/aortic body (reviewed in [28]). In the CNS, it is expressed in the sensory neurons of the nucleus tractus solitarius that receives signals from the epibranchial ganglia described above, in the area postrema, which senses toxins in the blood stream and cephalo-spinal fluid, and in the general visceral motor/pre-ganglionic neurons projecting to the parasympathetic and enteric ganglia, i.e., the dorsal motor nucleus of the vagus nerve, external formation of the nucleus ambiguus, and salivary nuclei. In addition to its expression in the visceral reflex circuitry, *Phox2b* is also expressed in neurons of the branchial/special visceral motor nucleus; the trigeminal nucleus (nV), the facial nucleus (nVII), the nucleus ambiguus, and the spinal accessory nucleus (nXI) [29].

The clinical importance of the RTN/pFRG was highlighted in studies that identified heterozygous mutations in *PHOX2B* in humans with congenital central hypoventilation syndrome (CCHS), a complex dysautonomic syndrome [31, 32]. CCHS typically manifests at birth by respiratory distress during sleep with no underlying muscular or cardiovascular defects and is frequently accompanied by a broad range of symptoms related to the sites of *PHOX2B* expression [33, 34].

Mice and rats are often regarded as interchangeable animal models in neuroscience research. Current analyses using transgenic and knockout mice for various targeting genes have added a wealth of scientific knowledge in neuroscience. However, other studies indicate that these two species differ in neuronal connectivity involved in common behaviors, such as spatial memory and recognition memory [35, 36]. In addition, rats are considered to be phylogenetically closer to primates than mice [37]. In this regard, several important findings related to respiratory neural network have so far been obtained mainly in rats. Moreover, we have observed that mice and rats have distinct features of the respiratory system both in anatomical composition and in mode of action (Onimaru and Ikeda, unpublished results). Based on these factors, further studies of the respiratory neural network in rats should be facilitated and developed. In this context, we describe here the generation of a bacterial artificial chromosome (BAC) transgenic (Tg) rat line, which harbors a mouse BAC carrying the *Phox2b* gene modified to drive enhanced yellow fluorescent protein (EYFP) and Cre recombinase-ER^T2^ (estrogen receptor T2) [38]. The resulting transgenic line showed EYFP expression in a pattern that mirrors that of endogenous *Phox2b* gene in rats and illustrated that the Tg rat is advantageous for investigation of the physiology of Pre-I neurons and the RTN/pFRG both *in vitro* and *in vitro*, and for identifying the CO_2_/O_2_ sensor molecules in the adult CNS and PNS.

## Materials and Methods

### Generation of *Phox2b*-EYFP/CreER^T2^ targeting construct and Tg rat

The *Phox2b*-EYFP-2A-CreER^T2^ Rec BAC construct was prepared by integrating a cassette composed of genes encoding a fluorescent protein and a CreER^T2^ [38], whose construct was kindly provided by Dr. P. Chambon at the University of Strasbourg, tethered by 2A peptide into clone 95M11 derived from the CHORI RP-24 C57BL/6J (B6) mouse genomic library using Red/ET recombination system (Gene Bridges, Heidelberg, Germany) [39]. The backbone of RP24-95M11 is a BAC clone and contains the entire mouse *Phox2b* gene (4,896 bp) with no other neighboring genes (total 198,590 bp, sequences 150,957 bp, sequences of upstream and downstream of the *Phox2b* 42,748 bp). Briefly, the insertion site of the cassette was just after the ATG of *Phox2b* coding region in exon1. All junctions were sequenced for validation. The BAC transgenic construct was prepared using Nucleobond Plasmid Purification kit (Macherey-Nagel, Düren, Germany) and purified for microinjection with a slightly modified procedure described by Abe [40] and the linearized BAC construct was used for microinjection. The 2A self-cleaving peptide is reported to mediate efficient generation of individual proteins [41]. Two different rat lines, which harbor tandemly-arrayed 30 copies of clones judged from the intensity of the band signals by southern blotting, were analyzed (1–10 and 4–10) for further studies. The line named W-Tg(Phox2b-EYFP/CreER^T2^)Keii is hereafter referred to as the Phox2b-EYFP/CreER^T2^. The establishment of another Tg rat line named W;LE-Tg(Gt(ROSA)26Sor/CAG-tdTomato)24Jfhy is described in [42]. In brief, this line contains the CAG promoter, loxP-flanked neomycin, and the red fluorescent protein (tdTomato) gene cassette, inserted into mouse ROSA26 locus BAC clone of RP23-244D9 and is hereafter referred to as ROSA26-tdTomato.

This study was carried out in strict accordance with the recommendations set in the Guide for the Care and Use of Laboratory Animals of the National Institutes of Health. The experimental protocols were approved by the Institutional Animal Care and Use Committee of Showa University (Permit Number: 53009, 54004), which operates in accordance with the Japanese Government for the care and use of laboratory animals. Newborn rats were deeply anesthetized with isoflurane in a glass bottle until nociceptive reflexes induced by tail pinch were completely abolished. The brainstem and upper spinal cord were isolated as described previously [4]. For the preparation of embryos, pregnant female rats were deeply anesthetized with isoflurane until vital signs were completely abolished, then caesarean section was performed to obtain fetuses at embryonic age (E)12.5 and E14.5.

### Genotyping

Genomic DNA was isolated from the ear punches or tail snips and dissolved in the direct PCR lysis reagent (Viagen Biotech Inc., Los Angeles, CA) with 0.5 mg/ml Proteinase K at 55°C for more than 3 hrs. After deactivation of proteinase K by heating at 85°C for 45 min, standard PCR reactions were performed using the following primers: 5'- GCTACGGCCTGCAGTGCTTCGCCC and 5'-CGGCGAGCTGCACGCTGCCGTCCTCG for the Phox2b-EYFP/CreER^T2^; 5'-CAAGAAGCCCGTGCAACTG (tdTomato-552F) and 5'-CCTCGTTGTGGGAGGTGATG (tdTomato-572F) for the ROSA26-tdTomato.

### Genomic sequence analysis

The genomic sequences containing *Phox2b* and flanking *Limch1* and *Tmem3* genes were downloaded from Ensembl: mouse (chromosome:GRCm38:5:66826857–67366858, Jan 2012), rat (chromosome:Rnor_5.0:14:42242139–42782140, Mar 2012), human (chromosome:GRCh38:4:41476526–42016527, Dec 2013), opossum (chromosome:BROADO5:5:182156202–18269620, Oct 2006), chick (chromosome:Galgal4:4:67779189–68319190, Nov 2011), painted turtle (scaffold:ChrPicBel3.0.1:JH584715.1–3069763:3609764, Dec 2011), *Xenopus* (scaffold:JGI_4.2:GL172695.1:1834960–2374961, Nov 2009), coelacanth (scaffold:LatCha1:JH127125.1:1–540002, Sep 2011), spotted gar (chromosome:LepOcu1:LG4:61957175–62497176, Dec 2011), and zebrafish (chromosome:Zv9:14:14629242–15169243, Dec 2008). Global pairwise alignment of the above sequences was carried out using shuffle-LAGAN [43], and the results were visualized using the VISTA Browser [44]. Annotation data were also obtained from Ensembl.

### Dissection of embryo and fetus

Staged rat embryos were obtained from timed pregnancies. The whole embryo/fetus was fixed in 4% paraformaldehyde/0.1 M phosphate buffered saline (PBS, pH 7.4) at 4°C for 3 hr and immersed in PBS or 18% sucrose/PBS. Images of the embryos and microdissected fetal organs (CNS and enteric gut) were obtained using a fluorescent stereoscopic microscope (model SZX16, Olympus, Japan). Hindbrains from embryos were dissected, and then the dorsal midline was cut to make an open-book preparation, as described previously [45]. The dissected hindbrain was placed on a glass slide and flattened by covering the ventricular surface with a cover slip for examination under a microscope (models BX51 and SZX16, Olympus).

### Immunofluorescence and three dimensional reconstitution

Immunofluorescence was carried out on tissue sections from rat fetuses essentially as described previously [46]. The samples were immersed in 18% sucrose/PBS, embedded in Tissue-Tek optimal cutting temperature (OCT) compound (Sakura Finetek, Torrance, CA), then frozen on dry ice, and cut into 16–20-μm-thick sections, followed by immunofluorescence. After drying for 30 min at room temperature, the sections were immersed in NT buffer (100 mM Tris(hydroxymethyl)aminomethane-HCl, 150 mM NaCl, pH 7.5 at 23°C) for 15 min. After repeating the immersion process three times, the sections were immersed in blocking buffer (1.5% Blocking Reagent (Roche Diagnostics Co. Indianapolis, IN) in NTT buffer (0.1% Triton X-100 (Sigma-Aldrich, St. Louis, MO) in NT buffer) for 60 min at 23°C and followed by primary antibody reaction for 6 hr at 23°C. After washing the sections in NTT buffer for 15 min three times, the secondary antibody reaction was performed for 1 hr at 23°C. After washing the sections in NTT buffer for 15 min three times, nuclear staining was performed. The sections were mounted using Vectashield mounting medium (Vector Laboratories, Burlingame, CA). The following primary antibodies were used in immunofluorescence: anti-PHOX2B (dilution, 1:2000 [22]), anti-tyrosine hydroxylase (TH) (dilution, 1:1000, Chemicon International Inc./Merck Millipore, Temecula, CA), and anti-VEGFR2/Flk-1 (dilution, 1:1000, R&D systems, Minneapolis, MN). The secondary antibodies for fluorescent staining (dilution, 1:2000) were Alexa Fluor 633 anti-guinea pig, Alexa Fluor 546 anti-rabbit, and Alexa Fluor 546 anti-goat (Molecular Probes/Invitrogen, Eugene, OR). 4,6-diamidino-2-phenylindole (DAPI, Sigma-Aldrich) was used for nuclear staining. Images of immunofluorescence samples were obtained with the 10× or 20× objectives of an Olympus FV1000 confocal microscope (Olympus) or the 10× objectives of a BX51 fluorescent microscope (Olympus). In each figure of these sections, the top is the dorsal side. Experiments were performed several times (as described in the Results) using different embryos or fetuses and the results were reproducible in different samples. Representative results are shown in the figures. Three-dimensional (3D) reconstruction from multiple images was performed as described previously [47] by Maxnet Co. (Fukuoka, Japan).

### Whole mount *in situ* hybridization

Whole mount *in situ* hybridization was performed on E12.5 embryos (n = 6) using single-stranded antisense digoxigenin-UTP (Roche)-labeled riboprobes, as described previously [46]. Hybridization was performed at 65°C. Signals were detected with an anti-digoxigenin antibody conjugated alkaline phosphatase (Roche) and 4-nitro blue tetrazolium chloride/5-bromo-4-chloro-3-indolyl-phosphate stock solution (Roche) for chromogen. The cDNA of mouse *Phox2b* was kindly provided by Drs. Christo Goridis and Jean-François Brunet (CNRS, Paris, France) and used as a template for the digoxigenin-riboprobe. The mRNA sequences covered by the probe are 92.2% homologous between rat and mouse and thus the use of the mouse probe enables detection of the rat mRNA. Experiments were performed several times using different embryos and the results were reproducible.

### Electrophysiology

Newborn rat brainstem-spinal cord preparations were used in electrophysiological studies (n = 5). Inspiratory activity corresponding to phrenic nerve activity was monitored from the fourth cervical ventral root (C4). Membrane potentials and input resistances of neurons in the pFRG were recorded by a blind whole-cell patch-clamp method [4]. The experiments were essentially performed as described previously [22, 23, 26] with the use of 0.1% sulforhodamine 101 acid chloride (Texas Red) (Sigma-Aldrich), instead of Lucifer Yellow. Tetrodotoxin (TTX), selective blocker of Na^+^ channel, was purchased from Sigma-Aldrich.

### Characterization of Cre-induced floxed fetuses

To determine the efficiency of Cre recombination, the Phox2b-EYFP/CreER^T2^ female rats were mated with the ROSA26-tdTomato male rats. Embryonic ages were based on the time of appearance of a vaginal plug with the first day being designated as E0.5. Tamoxifen (T-5648, Sigma-Aldrich) was prepared as 10 mg/ml stock solution in corn oil (C-8267, Sigma-Aldrich). Then 1.5 mg of tamoxifen/100 g of body weight was injected intraperitoneally into the pregnant mother for 2–3 consecutive days before delivery and fetuses were collected. It is noteworthy that tamoxifen injection resulted in 1–2 days premature birth in pregnant rats for unknown reason.

## Results

### Comparison of evolutionarily conserved non-coding sequences (CNSs) around the *Phox2b* rat gene

**Fig 1:**
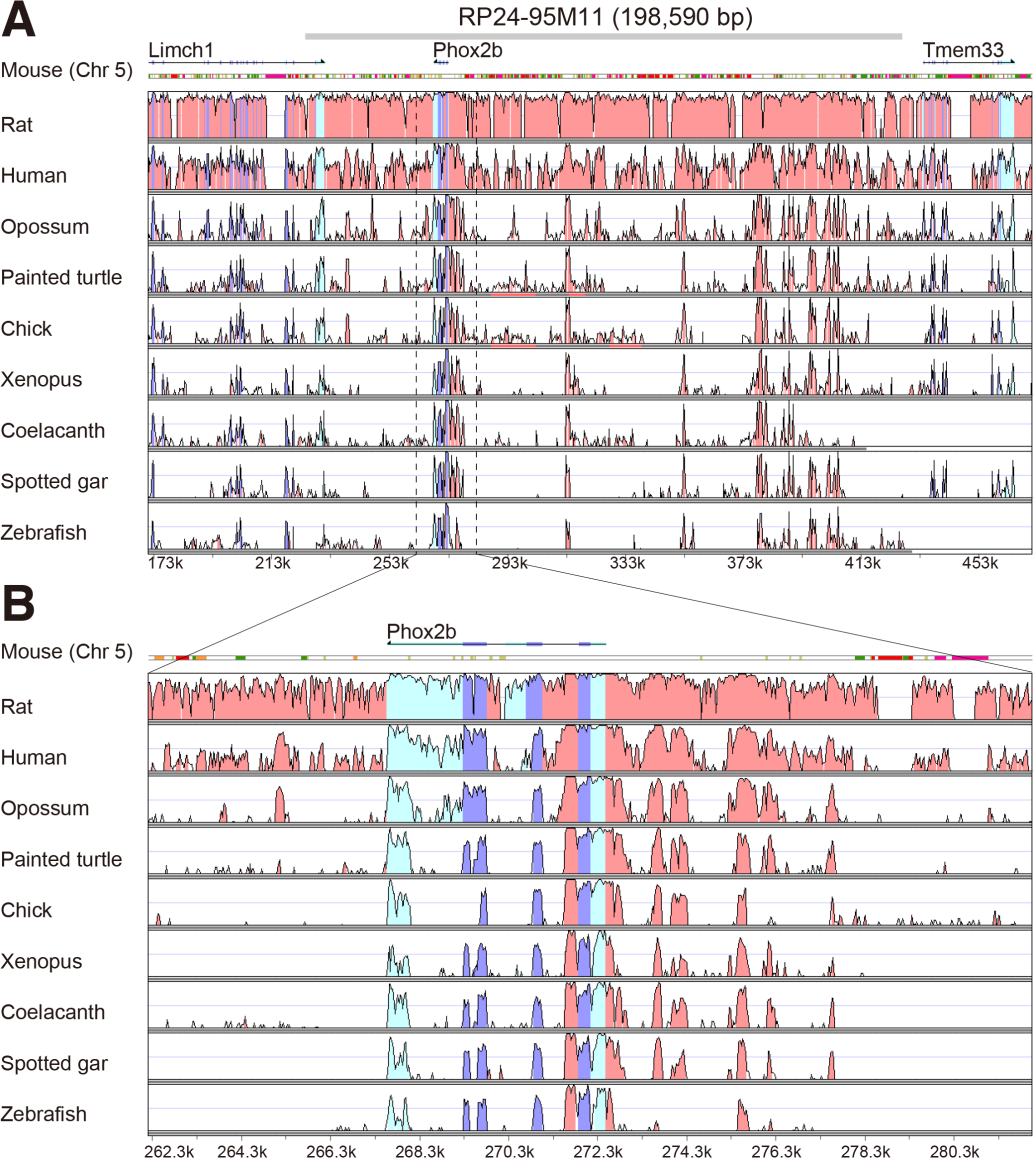
Position of conserved non-coding sequences around *Phox2b* exons. A, B, The VISTA plot of approximately 300 kb (A) span around the mouse *Phox2b* gene and the 20 kb (B) region closest to the gene are shown. The plot shows conserved sequences between mouse and rat, human, opossum, chicken, painted turtle, *Xenopus*, coelacanth, spotted gar, and zebrafish. *Abscissa*: mouse sequence, *ordinate*: percent identity in a 50-bp window. The conserved regions above the level of 60%/50 bp are highlighted under the curve, with pink indicating conserved non-coding sequence (CNS), blue indicating conserved exon, and cyan indicating untranslated region. The approximate position of the mouse BAC clone RP24-95M11 is shown in grey at the top of panel A. In A and B, numbers at the bottom of the plot indicate positions relative to the analyzed 540-kb mouse genome fragment (chromosome:GRCm38:5:66826857 (set as position 1)-67366858).

The unique expression pattern of *Phox2b* has been reported in mice, zebrafish, and lamprey during embryonic development [29, 30, 48–51], and in adult rat brain [13, 17]. Furthermore, the clinical symptoms of disease caused by mutations in the coding sequence of human *PHOX2B* are consistent with the expression patterns observed in rodents [31]. Previous studies reported that enhancers critical for temporal and spatial expressions of genes during development are frequently conserved among species [52]. In this regard, the transgenic mouse line that exploits a *Phox2b*-containing BAC (RP24-95M11) containing the *Phox2b* coding region together with upstream and downstream sequences in mice was confirmed to mirror the endogenous *Phox2b* expression pattern [53–55]. Since evaluation of the RP24-95M11 BAC clone could be useful for driving expression in transgenic rats, we compared the evolutionarily conserved non-coding sequences (CNSs) surrounding the *Phox2b* exons in mammals, together with related species, such as painted turtle, chicken, *Xenopus*, coelacanth, spotted gar, and zebrafish using mVISTA (Fig 1). The results showed that some CNSs were conserved only in placental mammals (rat, mouse, and human; Fig 1A). The flanking sequence of the mouse *Phox2b* gene showed remarkable resemblance to that of the rat *Phox2b* gene. The fact that RP24-95M11 BAC clone contained all the highly conserved regions between mouse and rat encouraged us to use the RP24-95M11 BAC clone in a transgenic rat model.

### Generation of *Phox2b*-EYFP/CreER^T2^ rat line

**Fig 2:**
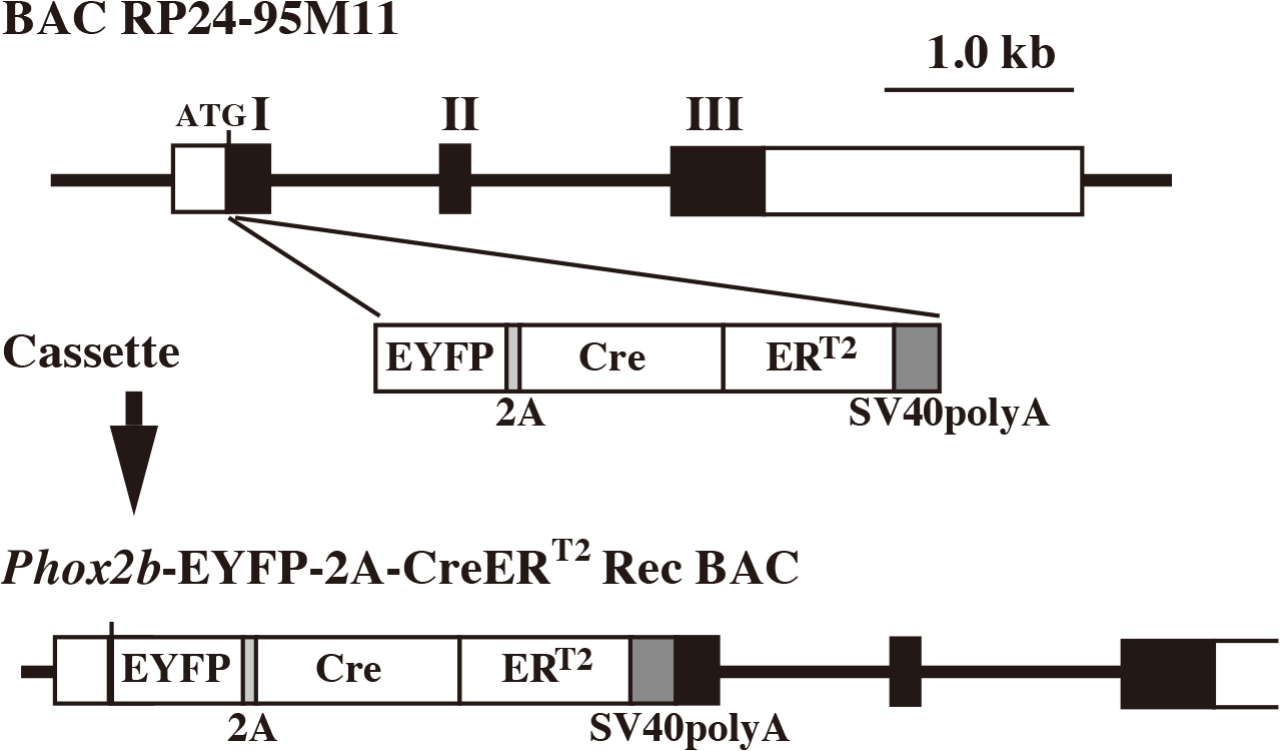
Schematic structure of the *Phox2b*-EYFP-2A-CreER^T2^ Rec BAC transgene. The exon-intron structure of the *Phox2b* gene is shown in the top section. The first exon which contains noncoding (white rectangle) and coding (black rectangle) regions, the second exon (black rectangle), and the third exon which contains coding (black rectangle) and noncoding (white rectangle) regions, are indicated as I, II, and III, respectively. The recombination strategy targeted the first exon (around the ATG site of *Phox2b* gene) inserting a cassette containing the enhanced yellow fluorescent protein (EYFP), 2A peptide coding sequence with fusion of Cre recombinase plus a modified tamoxifen-inducible estrogen receptor (CreER^**T2**^) sequence, and the SV40 polyadenylation sequence.

The *Phox2b*-EYFP-2A-CreER^T2^ Rec BAC construct was prepared by integrating coding sequences for a fluorescent protein and a CreER^T2^ tethered by 2A peptide into clone 95M11 derived from the CHORI RP24 C57BL/6J (B6) mouse genomic library (Fig 2). The backbone of RP24-95M11 is a BAC clone and contains the entire mouse *Phox2b* gene locus with no other neighboring genes (Fig 1). The RP24-95M11 BAC clone had been used previously to recapitulate the expression of the endogenous *Phox2b* gene in transgenic mice [53]. Because of the high homology between the mouse and rat *Phox2b* gene that extended into associated flanking regions, we expected that the RP24-95M11 BAC contained sufficient regulatory regions required to confer expression patterns of *Phox2b* gene in the rat during development and in adults. Similar findings were observed in two different lines that harbor tandemly-arrayed 30 copies of the clones.

### *Phox2b*-EYFP reporter is expressed at sites of endogenous PHOX2B in developing rat embryos

**Fig 3:**
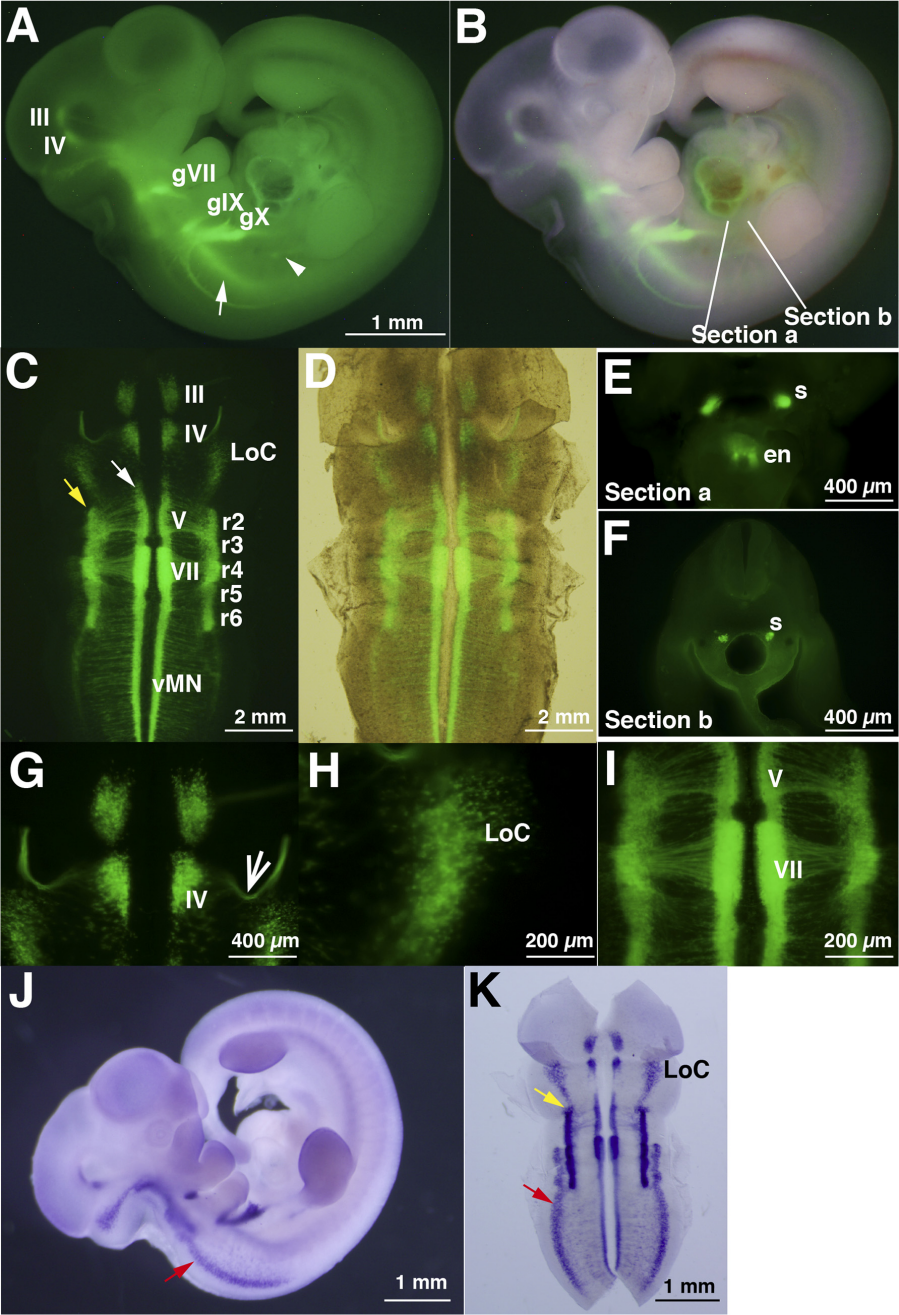
EYFP expression from the Phox2b-EYFP/CreER^T2^ Tg is comparable to *in situ* hybridization patterns of endogenous *Phox2b* in E12.5 rat embryos. A, The transgenic (Tg) rat embryo at E12.5 (somite number 39) harboring the *Phox2b*-EYFP-2A-Cre/ER^**T2**^ Rec BAC transgene exhibits fluorescence in known sites of *Phox2b*/PHOX2B expression. The EYFP signal is detected in the oculomotor (III) and trochlear (IV) motor nuclei in the central nervous system (III and IV), and in the three epibranchial sensory/distal ganglia; geniculate VIIth, petrosal IXth, and nodose Xth ganglia (gVII, gIX, and gX). Primary sympathetic chain (arrowhead) and neurons in the ventral columns of neural tubes are also positive (arrows). B, Superposition of fluorescence from A on bright field image. The positions of coronal sections are indicated as "Section a" in E and "Section b" in F. C, Flat-mount preparation of an E12.5 fetal rat midbrain and hindbrain shows transgene expression. In the mesencephalic region, the signals are found in the oculomotor (III) and trochlear (IV) motor nuclei and in the forming locus coeruleus (LoC). In the rhombencephalon, the signals are found in the ventral stripe composed of V, VII, and ventral motor neurons (vMN) and in the lateral stripe of rhombomeres 2, 3, 4, 5, and 6 (r2, r3, r4, r5, and r6, respectively). D, Superposition of figures under white-light and fluorescence of C. E, F, Coronal sections at the level of sections taken at the Sections a and b shown in B. The sympathetic ganglionic chain (s) and enteric nervous system (en) are fluorescent-positive. G-I, Magnified figures in C. The unique projection of axons from the trochlear nucleus is indicated by the sharp arrow. J, Whole-mount *in situ* hybridization of rat embryo at E12.5. K, Flat-mount preparation of midbrain and hindbrain of the embryo shown in J.

To examine whether EYFP fluorescence driven by *Phox2b*-EYFP/CreER^T2^ transgene conferred expression of endogenous *Phox2b* in the developmental nervous system, we examined the fluorescence of the whole embryo at E12.5 (n = 6, somite number 38–40) (Fig 3A and 3B). Fluorescent signals were found in the oculomotor (III) and trochlear (IV) motor nuclei (Fig 3A, III and IV), as reported previously in the developing "mouse" mRNA/protein [29]. Signals were found in the epibranchial distal VIIth, IXth, and Xth ganglia (Fig 3A, gVII, gIX, and gX). Signals were also detected in primary sympathetic chain in the whole embryo (arrowhead in Fig 3A), as well as thick coronal sections cut at the level of Sections a and b in Fig 3E and 3F (s, sympathetic chain). Progenitors of the enteric nervous system were also fluorescent-positive (Fig 3E, en). We confirmed the region of fluorescent signals in flat-mounted hindbrains of the embryos (Fig 3C and 3D). Fluorescent signals in the hindbrain were organized in the longitudinal columns, in agreement with mouse *Phox2b* mRNA expression [29]. Branchial motor neurons (V and VII in Fig 3C) and visceral motor neurons (vMN in Fig 3C), which reside in the ventral columns (white arrows in Fig 3A and 3C), were clearly identified. A unique dorsal projection of trochlear (IV) axons was also identified along the midbrain-hindbrain boundary (arrow in Fig 3G). The axons of V and VII motor neurons converged towards the future exit points of their cranial nerves (Fig 3C and 3I). The locus coeruleus (LoC), the location of the major noradrenergic neuronal population in the brain, situated in the rostral hindbrain, was also fluorescent-positive at this stage (Fig 3C and 3H), consistent with the fact that PHOX2B is an upstream transcription factor for *dopamine-ß-hydroxylase* gene [30].

Using embryos at the same stage of E12.5, we performed whole-mount *in situ* hybridization using *Phox2b* riboprobe and confirmed the fluorescent signals traced *Phox2b* endogenous expression followed by the flat-mount preparation (Fig 3J and 3K) (n = 3). In general, the fluorescent signals were highly similar to those of endogenous mRNA expression in E12.5 embryos, although the signals in the dorsal stripe were more apparent in endogenous expression than in fluorescence (red arrows in Fig 3J and 3K). The lateral stripe (yellow arrows in Fig 3C and 3K) was also similar. The dorsal and lateral stripes contained cells seen in the mantle layers of mice at comparable stage (Figs 2 and 5 in [29]).

**Fig 4:**
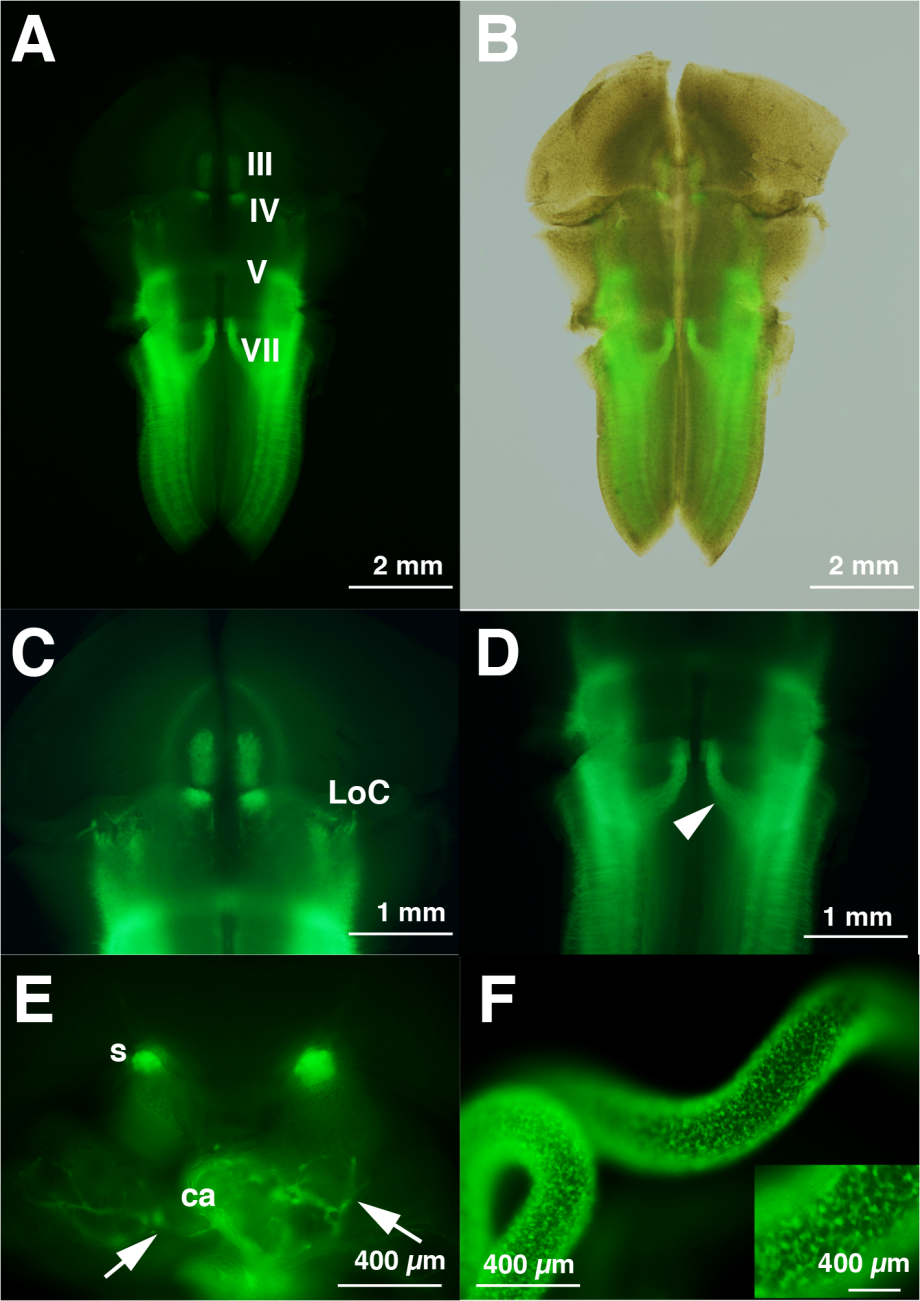
EYFP fluorescence is evident in known sites of *Phox2b*/PHOX2B expression among E14.5 Phox2b-EYFP/CreER^T2^ Tg rat embryos. A, C, D, Flat-mount preparation of the midbrain and hindbrain of fluorescent-positive E14.5 embryo. The signals in the III and IV motor nuclei and LoC are weaker than those of E12.5 shown in Fig 3. The dorsal migratory trajectories of motor neurons of V and VII are clearly evident in D (arrowhead). B, Superposition of figures under white-light and fluorescence of A. E, Coronal thick section (~100 μm) at the level of cardiac primodia (ca). Axons of the vagus (Xth) enter the developing cardiac primodia (arrows). The sympathetic ganglionic chain (s) are fluorescent-positive. F, Fluorescent-positive enteric nervous system progenitors derived from enteric neural crest cells are seen in the rat E14.5 intestine. The inset shows magnified views of the intestine.

We also examined the E14.5 Tg embryos (n = 3). The signals in the III and IV motor nuclei and the locus coeruleus were still found but became weaker than those of E12.5 (Fig 4A, 4B and 4C; III, IV, LoC). Medially branched signals, which should reflect dorsal migratory trajectories of motor neurons of V and VII, were clearly observed; neurons of VII born in r4 (Fig 3C) migrated caudally through r5/r6 and turned dorsally (arrowhead in Fig 4D). Axons of the Xth motor neurons (arrows in Fig 4E) that entered into the developing cardiac primodia (Fig 4E, ca) were fluorescent-positive. The sympathetic chain was also fluorescent-positive (Fig 4E, s)

In mice, PHOX2B is a marker of all neural crest-derived cells within the developing gut [56]. The enteric nervous systems derived from the vagal and sacral neural crest cells (sometimes called as ENCCs) were fluorescent-positive in the intestine of Tg rat at E14.5 (Fig 4F).

### EYFP from *Phox2b* Tg is expressed in respiratory centers of neonatal rats

Pre-I neurons show pre-/post-inspiratory bursts that are typically interrupted by inspiratory-related inhibition, and they constitute one of the respiratory rhythm generators in the medulla of the newborn rat [4, 57]. We reported previously that Pre-I neurons of the pFRG are authentically CO_2_-sensitive [58] and that the expression of PHOX2B in Pre-I neurons correlated well with CO_2_/H^+^ sensitivity in neonatal rats (reviewed in [59]). In adult rats, the RTN is considered the CO_2_/pH sensor system [12, 13]. PHOX2B is also expressed in the embryonic mouse parafacial population of rhythmically bursting neurons, which may correspond to pFRG in neonatal rats [60]. Moreover, the *Phox2b* ^*27Ala/+*^ mouse, a model of CCHS for the most common human mutation of expanded alanine stretch, does not respond to hypercapnia and dies at birth from respiratory failure [61]. These results suggest that *Phox2b* is required for production of chemosensor-rhythm generator neurons. Therefore, fluorescent-labeling of cells can be of great use to identify Pre-I neurons, which number around 1,000 per one side in the postnatal day (P)0-P1 fetal rat (Ikeda and Onimaru, unpublished results), as determined during electrophysiological experiments using freshly prepared samples.

**Fig 5:**
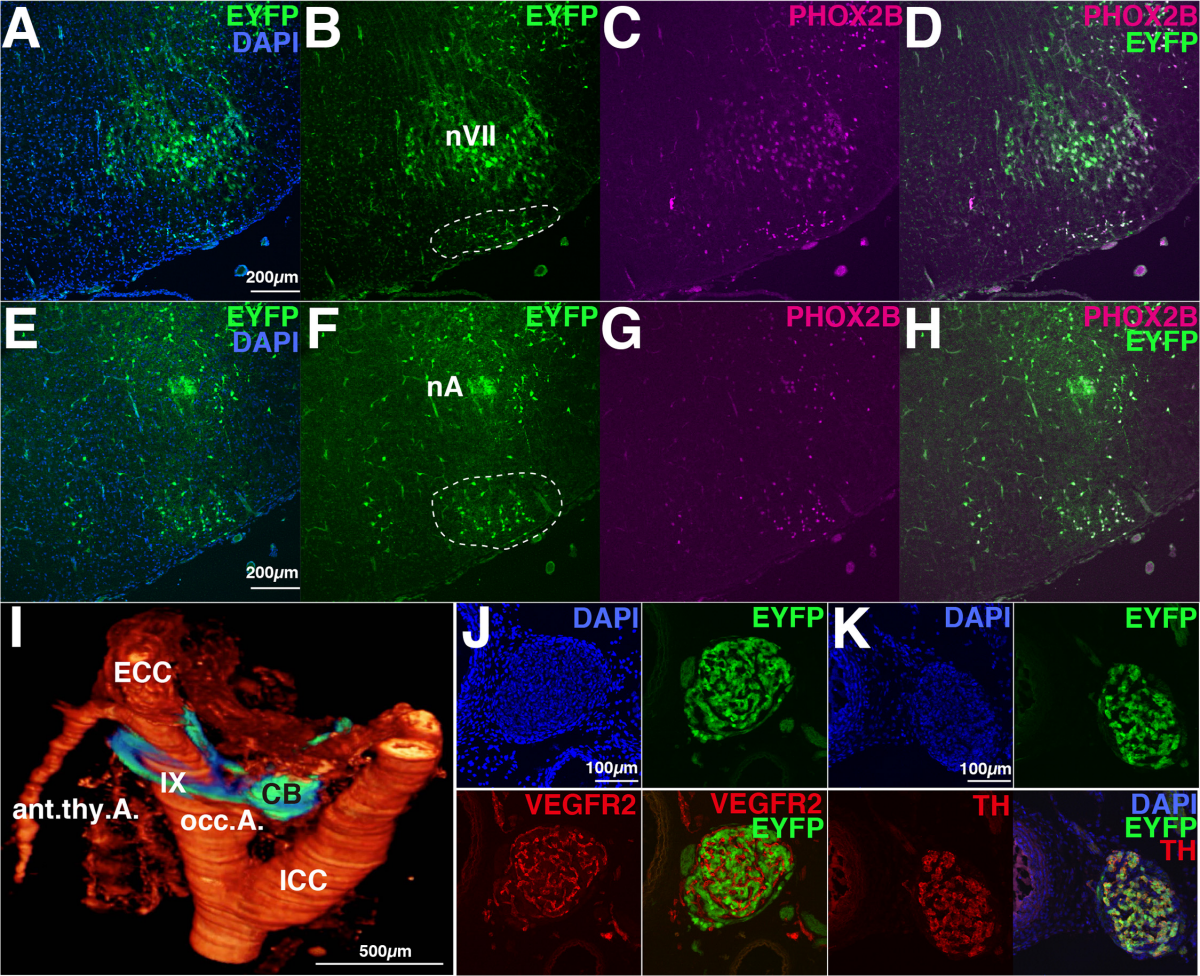
Expression of the EYFP reporter in respiratory associated neurons of Phox2b-EYFP/CreER^T2^ Tg neonatal rats. A-H, Coronal sections in retrotrapezoid nucleus (RTN)/parafacial respiratory group (pFRG) of the medulla. A-D, The rostral part of RTN/pFRG which resides in the ventral surface of the facial nucleus (nVII) are fluorescent-positive (A-D). Most neurons of the facial nucleus are also fluorescent-positive but their signals vary depending on the cell groups. E-H, The caudal part of RTN/pFRG resides in the ventral and medial sides of the nucleus ambiguus (nA), and is also fluorescent-positive. All EYFP-positive cells are immunoreactive for anti-PHOX2B (C, D, G, and H). I-K, The carotid body (CB) of the Tg rat is EYFP-positive. I, Three-dimensional reconstitution using complete serial sections of surrounding CB tissues. ECC, external carotid artery; ICC, internal carotid artery; IX, glossopharyngeal nerve; occ. A., occipital artery; ant. thy. A., anterior thymic artery. J, Coronal section of the rostral part of CB stained with anti-VEGFR2 (capillary endothelial cell marker) and DAPI. K, Coronal section of the rostral part of CB is immunopositive for tyrosine hyroxylase (TH).

As described above, the Pre-I neurons are distributed in the relatively narrow region of the rostral medulla named pFRG. Two major neuronal cluster regions of pFRG have been identified; the rostral pFRG and the caudal pFRG (Fig 1 in [22]). We examined whether the distribution pattern of EYFP-positive signals in the rostral medulla to caudal pons in the newborn Tg rat was similar to that of previously-reported endogenous PHOX2B-immunoreactive cells (n = 9). The cells of rostral pFRG resided in the superficial area just ventral to the facial nucleus (nVII) and in close proximity to the surface (Fig 5A–5D, a dotted white circle in B). The cells of caudal pFRG resided ventral to the nucleus ambiguus (nA) at the end of the nVII (Fig 5E–5H, dotted white circle in F). In Tg P0-P1 fetuses, the EYFP-positive cells were also distributed in the area postrema, the nucleus of the solitary tract, and the dorsal motor of the vagus (data not shown), in which others have reported previously the expression of endogenous PHOX2B in rodents (reviewed in [28].

The carotid body is the main arterial chemoreceptor organ sensitive to the changes in blood O_2_, CO_2_, and pH levels and plays crucial roles in the regulation of respiration. The carotid body is located at the carotid artery bifurcation and expresses *Phox2b* (reviewed in [28]). To determine the position of the carotid body relative to the surrounding arteries, we performed 3D-reconstruction using complete serial sections of the Tg rat carotid body tissues (Fig 5I). The carotid body in rats was attached to the occipital branch of the external carotid artery (occ. A.). The glomus type I cells in the carotid body are known to be synaptically connected to the nerve terminals of petrosal (IX) ganglion neurons. The IXth axons were also fluorescent positive. Sections of the carotid bodies (n = 3) were also immunostained. As expected, abundant blood capillaries were identified in the carotid body, which were stained by anti-VEGFR2 (vascular endothelial growth factor receptor 2) (Fig 5J, red). In response to low O_2_, glomus type I cells release various transmitters, such as dopamine and noradrenaline [62, 63]. We confirmed that the carotid body was positive for tyrosine hydroxylase (TH) together with EYFP fluorescence (Fig 5K, red and green)[64], consistent with the fact that *Phox2b* is required for the expression of two enzymes (*tyrosine hydroxylase* and *dopamine-ß-hydroxylase*) in the catecholamine biosynthesis pathway.

### PHOX2B immunoreactivity and CO_2_ sensitivity of EYFP-positive Pre-I neuron in RTN/pFRG

**Fig 6:**
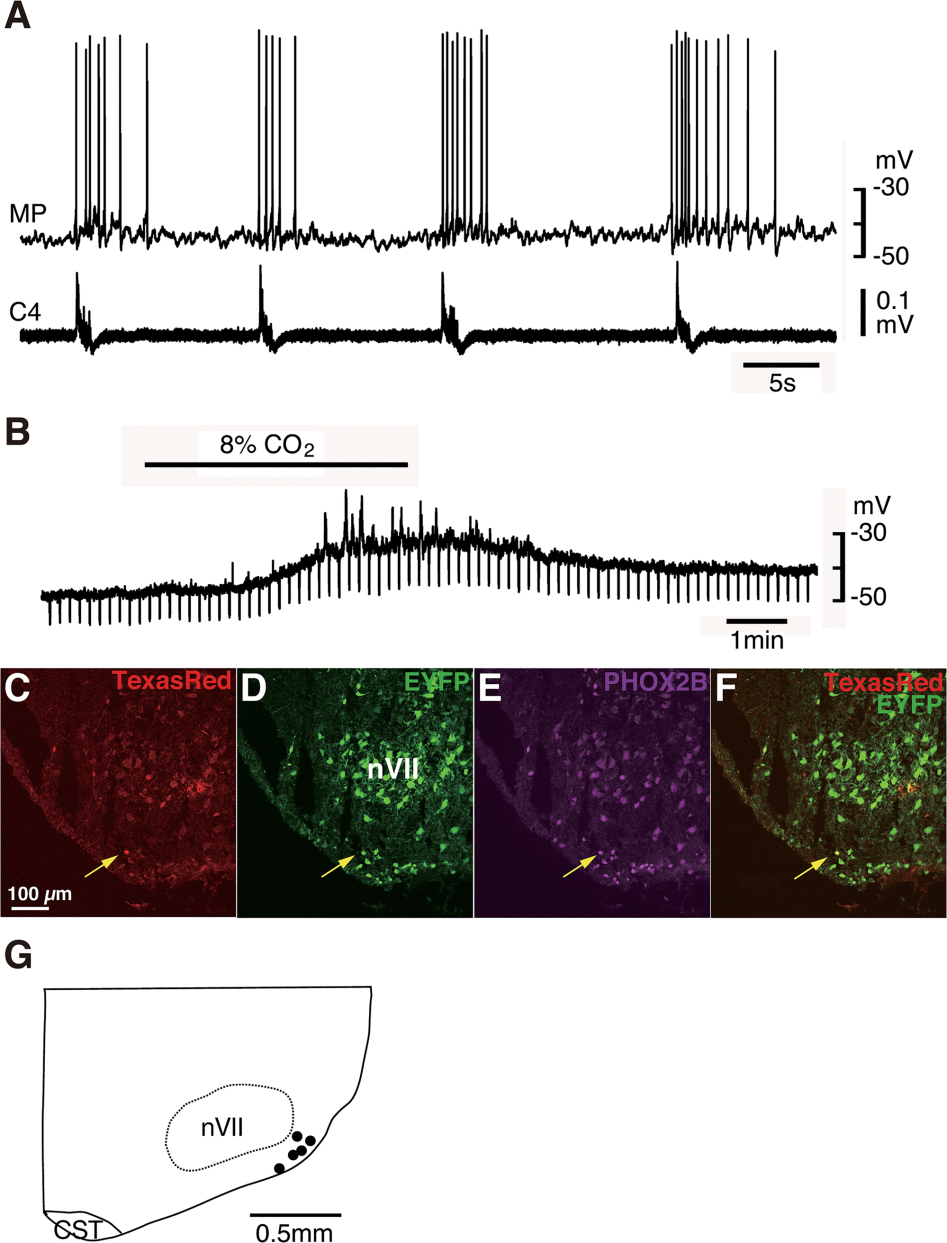
Pre-I neurons in Phox2b-EYFP/CreER^T2^ Tg rats are EYFP-positive. A, Burst pattern of the recorded neuron in the rostral pFRG of newborn Tg rat shows typical Pre-I neuron discharge. MP, membrane potential; C4, fourth cervical ventral root activity. B, Membrane potential response to high CO_2_ stimulation (2% CO_2_ → 8% CO_2_) in the presence of tetrodotoxin (TTX). Negative deflections of the baseline membrane potential are proportional to input resistance, indicating that the neuron is CO_2_ sensitive. C-F, Coronal sections of brainstem-spinal cord preparation after electrophysiological analysis (A, B) to identify the location of the recorded neuron stained by Texas Red (yellow arrows). The recorded neuron is EYFP-positive and PHOX2B-immunoreactive. G, Distribution of EYFP-positive Pre-I neurons with CO_2_ sensitivity, plotted in the corresponding slice at the level of 500–600 μm rostral to the caudal end of the facial nucleus. Each dot represents a single recorded neuron (n = 5). nVII, facial nucleus; CST, corticospinal tract.

We reported previously that the majority of Pre-I neurons in the rostral pFRG were PHOX2B positive and that the PHOX2B-positive Pre-I neurons in neonatal rat were glutamatergic and expressed neurokinin 1 receptor (NK1R) [22, 59] (data not shown), consistent with previous reports by other investigators [12, 13, 65]. To investigate whether the EYFP-positive cells in the pFRG of this novel Tg rat indeed show the characteristics of the Pre-I neurons, we performed whole-cell patch clamp recordings from EYFP-positive cells in the region of the rostral pFRG of the neonatal rat (n = 5). The average resting membrane potential was -50.0±2.6 mV with an input resistance of 783±203 MΩ in standard solution (5% CO_2_) (Fig 5A). The neurons were depolarized in response to hypercapnia (2% → 8%) in the presence of 0.5 μM TTX, accompanied by an increase in input resistance (Fig 6B) (2% CO_2_: -46.8 ± 4.3 mV, 374 ± 141 MΩ, 8% CO_2_: -38.8 ± 6.5 mV (*P*<0.05), 537 ± 237 MΩ (*P*<0.05), respectively). These results indicate that the mechanism of the neuronal CO_2_ response was postsynaptic and probably mediated by closing K^+^ channels, as discussed previously [23, 58]. These neurons were marked by Texas Red during recording. After recording, we confirmed that they were immunoreactive with PHOX2B (Fig 6C–6F). The results indicate that the EYFP-positive cells in the pFRG have the characteristic of Pre-I neurons. We mapped the distribution of CO_2_-sensitive EYFP-positive Pre-I neurons in five preparations (Fig 6G). The results showed dense localization of these neurons in the superficial area just ventral to the facial nucleus, as described previously [22].

### Induction of CreER^T2^ in *Phox2b* BAC Tg fetal rats

**Fig 7:**
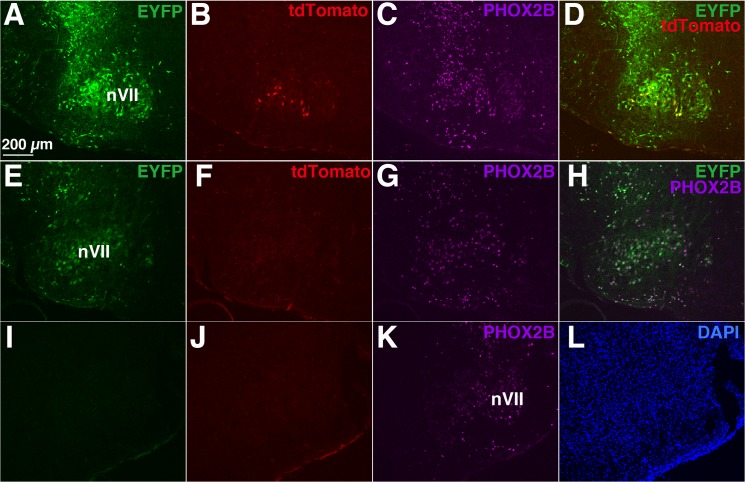
Analysis of CreER^T2^ recombinase activity. A-D, Cre recombinase activity induced by estrogen analog tamoxifen (CreER^**T2**^, estrogen receptor T2, [38]) was examined by fluorescence in fetus harboring both the *Phox2b*-EYFP-2A-CreER^**T2**^ transgene and the ROSA26-tdTomato transgene born from pregnant rat treated with tamoxifen. The tdTomato red signals are detected in the facial nucleus (nVII). E-H, No red fluorescence is present in fetus harboring both the *Phox2b*-EYFP-2A-CreER^**T2**^ transgene and the ROSA26-tdTomato transgene born from pregnant rat not treated with tamoxifen. I-L, The absence of red fluorescence in the fetus harboring only the ROSA26-tdTomato transgene born from pregnant rat treated with tamoxifen. C, G, and K, Immunofluorescence using anti-PHOX2B antibody. P0-P2 fetuses are used. For EYFP excitation (panels A, E, and I), a multi-argon laser is applied and emission bandwith is 500–530 nm. For tdTomato (panels B, F, and J) and AlexaFluor633 (panels C, G, and K) excitation, a helium-neon laser is used and emission bandwith is 560–620 nm and 650- nm, respectively. All images in the panels (A-L) were taken under the same condition on the same day.

Next, we investigated whether *Phox2b* promoter/enhancer induces efficient expression of EYFP and CreER^T2^. The transgenic construct (Fig 2) contained coding sequences for EYFP and CreER^T2^ separated by the 2A peptide, whose sequence caused ribosomal skipping during translation and subsequent cleavage of the translated polypeptide at the 2A site, resulting in the generation of distinct polypeptides that were subsequently folded independently into their native structures [66]. Theoretically, the inducible CreER^T2^ is active only in the presence of tamoxifen [38]. We used fetuses that harbored both the Phox2b-EYFP/CreER^T2^ transgene and the ROSA26-tdTomato reporter transgene obtained by crossing the two Tg rat lines as the parent. Upon administration of tamoxifen to mother Phox2b-EYFP/CreER^T2^ rats, tdTomato red signal was observed in the facial nucleus (nVII) of the fetus (Fig 7B) and those were EYFP-positive (n = 9 pups from 4 mothers) (Fig 7A and 7D). In the absence of tamoxifen, signals were not detected in the fetus that harbored both transgenes (n = 3 pups from 2 mothers) (Fig 7F).

Furthermore, fetuses of mothers not treated with tamoxifen (n = 3 pups from 1 mother) showed tdTomato signals in the form of non-overlapping expression with EYFP (data not shown). Although the exact reasons for the non-inducible expression of Cre recombinase has not yet been addressed, one possibility would be that the expression of CreER^T2^ at relatively high levels (30 copies in our rats) display Cre recombinase activity without tamoxifen, a similar phenomenon has already been reported in mice [67]. Expression of tdTomato was negative in fetuses with only the ROSA26-tdTomato transgene and lacking the Phox2b-EYFP/CreER^T2^ transgene (n = 3) (Fig 7I–7L). These findings indicate that the Cre recombinase was active under the control of *Phox2b* promoter/enhancer and inducible in the Phox2b-EYFP/CreER^T2^ rat line.

## Discussion

### *Phox2b* gene possesses evolutionarily conserved enhancers

Several studies have mapped the anatomic location of the central respiratory systems and peripheral gas sensing organs and determined their physiological properties both in rats and mice, in addition to the sea lamprey, zebrafish, and *Xenopus*. For example, the paratrigeminal respiratory group (pTRG) of lambreys brainstem is thought to play an important role in respiratory rhythm generation [68–71]. Furthermore, in *Xenopus lavies*, serotonin-immunoreactive (5-HT-IR) neuroepithelial cells located in the gills are O_2_ chemosensitive [72]. However, the relationship between *Phox2b* expression and primary CO_2_ sensing neuronal regions in the brain and its involvement in respiratory rhythm generation have not been investigated in animals other than the mouse and rat. Our cross-species comparisons of sequence at the *Phox2b* locus demonstrate that most of the conserved non-coding sequences (CNSs) are shared among gnathostomes. This observation suggests that the expression patterns of *Phox2b* should be conserved among these species, that the respiratory central neural system was probably established before radiation of gnathostomes, and that extant gnathostomes share a similar regulatory network, *i*.*e*., similar sensor molecules and neural connectivity through the conserved expression of at least *Phox2b*. In this context, identification of the *Phox2b* expression pattern in the central respiratory groups of neurons and in the primary chemoreceptor cells is of great significance because it is pre-requisite for this argument.

### CO_2_/H^+^ central chemosensitivity

It is generally accepted that increases in blood CO_2_ levels are detected mainly by chemosensors within the CNS and that CO_2_ central chemoreceptors play a critical role in respiratory and cardiovascular control. Although the primary sensor cells and their exact neural networks that control respiration and cardiac system remain elusive, various central chemoreceptor cells are known to regulate and/or modulate respiratory neural activity in the brain; including the RTN/pFRG, nucleus paragigantocellularis, parapyramidal region of raphé nuclei, locus coeruleus, area postrema, nucleus tractus solitarius, lateral hypothalamus, and the fastigial nucleus of the cerebellum [73–75]. The RTN/pFRG is especially important since CO_2_ chemosensitivity does not depend on presynaptic input [76]. In this regard, the PHOX2B-positive Pre-I neurons in the RTN/pFRG are CO_2_-sensitive under blockade of either Na^+^ channels or Ca^2+^ channels [22, 58, 59]. These observations strongly indicate that CO_2_ sensitivity is probably the intrinsic property of postsynaptic PHOX2B-positive Pre-I neurons [23]. Interestingly, Gourine and coworkers [77] used slice preparation of adult rats to argue that central chemoreception in the ventral medulla is mediated by ATP and its receptor. In another study, the same group also showed that a fall in local tissue pH results in increase in Ca^2+^ and release of ATP by astrocytes, but not neurons [78]. Using slice preparation of P7-12 rats, Wenker et al. [79] suggested that astrocytes in the RTN(/pFRG) sense CO_2_/H^+^ by inhibiting a Kir4.1-Kir5.1-like current through chemoreception by a purinergic mechanism. By applying multiscale Ca^2+^-sensitive dyes and imaging system for EYFP-labeled PHOX2B-positive cells, we can address this point by calculating the percentage of responding cells among PHOX2B (EYFP)-positive cells that show increased change of fluorescence (a direct measure of intracellular Ca^2+^ response) under 2% → 8% CO_2_ conditions. In other words, calcium imaging experiments of RTN/pFRG using this Tg rat can clarify the proportion of PHOX2B (EYFP)-positive cells that are indeed Pre-I neurons (Onimaru and Ikeda, unpublished results). Furthermore, the molecules responsible for sensing these changes among the Pre-I neurons and/or astrocyte remain elusive. This aspect will be discussed below.

### Relationship between RTN and pFRG

The RTN and ventral pFRG share a multitude of anatomical and functional similarities; however, the exact relationship between these two areas is still unclear. The Pre-I neurons display phasic Pre-I firing pattern [4], while RTN neurons of the adult rat do not [13, 15]. Ablation of PHOX2B-positive cells in the RTN/pFRG of the Phox2b-EYFP/CreER^T2^ Tg line in a restricted manner at appropriate days after birth, should enable studies to examine the issue of whether the pFRG and RTN correspond to neonatal and adult forms of the same neuronal population (see below). The availability of the Phox2b-EYFP/CreER^T2^ Tg will also permit analysis of another important issue of whether the RTN/pFRG is a major chemoreceptor site itself in the *adult* rat (under sleep and/or conscious state), or whether it primarily acts as the integration site that receives various inputs from other chemoreceptors in the brain, such as the medullary raphé, nucleus tractus solitarius, lateral hypothalamus, and peripheral sensory receptor cells [80]. In mice, the RTN/pFRG neurons originate from the dB2 (class B dorsal interneurons of the hindbrain) progenitor cells, which are identified by co-expression of *Phox2b*, *Lbx1*, *Egr2*, and *Atoh1* (reviewed in [81, 82]. Further lineage-tracing studies using the Phox2b-EYFP/CreER^T2^ Tg rat line are possible to identify and/or confirm the developmental origin and migratory pathway of RTN/pFRG in rat embryos during development.

### *Phox2b* as a key gene for cells harboring chemosensors

The PHOX2B-positive cells in the RTN/pFRG are essential for survival at least in early life of animals, because their loss in patients with CCHS or by genetic manipulation in mice is associated with blunted chemoreception and unstable breathing [61], although it should be noted that there may exist additional functional abnormalities in the peripheral sensory organs in these cases. The peripheral chemoreceptors, on the other hand, reside in the carotid body/aortic body, which is located at the bifurcation of the artery and detects changes in arterial blood O_2_ and glucose. The carotid body is also considered to be involved in CO_2_ chemoreception [83]. Our results showed PHOX2B expression and comparable localization of EYFP in the Phox2b-EYFP/CreER^T2^ Tg rat (Fig 5). The area postrema, which resides in the brainstem close to the nucleus tractus solitarius and senses toxins in the bloodstream [84], was also EYFP-positive (data not shown). Based on these observations, cells that detect changes in blood gases could commonly express the PHOX2B transcription factor. We propose that PHOX2B regulates the expression of gas sensor receptor molecules in a set of sensor cells located both in the CNS and the PNS. In this context, the Phox2b-EYFP/CreER^T2^ Tg rat labeled with EYFP could be suitable for isolating such sensory cells and identifying the molecules responsible not only for central CO_2_/H^+^ chemoreception, but also peripheral O_2_ chemoreception, such as the carotid body using microarrays, next generation DNA sequencing, and proteomics. Moreover, one can use the fluorescent-labeled intestine of this Tg rat for neural network analysis and identification of the main features of enteric neurons derived from the neural crest.

### Wide use of the Tg rat

Various mouse models are currently available for understanding the respiratory neural network, including a model for CCHS [18, 55, 61, 81, 85]. We believe that the Tg rat line described here, which is endogenously labeled with EYFP, adds another dimension to the previously available models because it can be used to identify Pre-I neurons of rat in electrophysiological studies. Candidate chemosensitive sensor molecules have also been reported. Extensive studies have shown that the TASK-2 channel (KCNK5, potassium channel subfamily K, member 5) is one such pH sensor molecule in the mouse brain, if not the main player [86, 87]. The transient receptor potential gene (*trp*) superfamily TRP proteins have recently been identified as gaseous messenger molecules, including O_2_, H_2_S, and CO_2_ in various tissues (reviewed in [88]). Among the *trp*, TRPA1 mediates hyperoxia- and hypoxia-induced cationic currents in vagal and sensory neurons [89]. In this context, the Phox2b-EYFP/CreER^T2^ Tg rat would be a powerful tool for identifying sensor molecules not only in the CNS (the RTN/pFRG and the area postrema), but also in the PNS, such as the carotid body/aortic body and the enteric neurons.

Apart from using EYFP fluorescence as a marker, the expression of inducible Cre in Pre-I neurons would be useful in anatomical, physiological, and pathophysiological studies on the respiratory neural network and enteric nervous system. The Cre-mediated recombination system (Cre/loxP) is currently the most commonly used method for deletion or ectopic expression of genes in a cell-type specific manner, with the temporal control of gene expression achieved by fusion of Cre recombinase to a modified tamoxifen-inducible estrogen receptor (CreER^T2^). We document that Cre recombinase was expressed in EYFP-positive cells under the control of *Phox2b* in our Tg rat. By crossing the Phox2b-Cre/ER^T2^ driver rat with the other Tg rats with floxed genes, it becomes possible to induce various genes under the control of *Phox2b* enhancer/promoter region with appropriate timing. For example, crossing the line with another transgenic rat line harboring a diphtheria toxin gene rendered transcriptionally silent by a floxed sequence, can activate the diphtheria toxin in the double (Cre and floxed diphtheria toxin constructs) Tg rat upon administration of tamoxifen and Cre-mediated excision of the floxed region. This temporal control permits specific ablation of Cre-expressing cells at appropriate developmental stages or postnatal rats. Of particular interest is the ablation of EYFP-positive cells in young and old adult rats, because this approach should address the following questions; 1) Is the RTN/pFRG also the key central respiratory rhythm generator neuron complex in the adult? During rat postnatal development, the period around P12-13 is critical since abrupt changes in ventilatory and electrophysiological pattern have been documented (reviewed in [90]). 2) What is the pathophysiology of late-onset CHS (LO-CHS), in which patients are symptom-free until at least 20 years of age? In addition, the Tg rat lines with floxed light activated various channels are also of great use for analysis of the respiratory neurocircuitry. The use of floxed viruses would be another choice. Previous studies in adult rats demonstrated that optogenetic excitation of neurons of the RTN/pFRG could induce active expiration and reset the respiratory rhythm in adult rats [91, 92]. These results are similar to those obtained in newborn rat preparations [93] and suggest that a structure corresponding to the neonatal pFRG exists in adulthood with some developmental changes.

In summary, apart from being a EYFP marker reporter, the expression of CreER^T2^ provides a useful tool for characterization of tissue-type, cell type- and time-specific functions of *Phox2b*/PHOX2B in developmental processes and diseases, as well as physiological functions in the fetus and adult rat, without the bias produced by non-specific side effects of systemic knockdown and overexpression of additional exogenous genes.
